# The Influence of Response Inhibition Training on Food Consumption and Implicit Attitudes toward Food among Female Restrained Eaters

**DOI:** 10.3390/nu12123609

**Published:** 2020-11-24

**Authors:** Noam Weinbach, Eldad Keha, Hila Leib, Eyal Kalanthroff

**Affiliations:** 1 School of Psychological Sciences, University of Haifa, Haifa 3498838, Israel; 2 Department of Psychology, The Hebrew University of Jerusalem, Jerusalem 9190501, Israel; eldadkeha@gmail.com (E.K.); hilaleib326@gmail.com (H.L.); 3 Department of Psychology, Achva Academic College, Arugot 7980400, Israel

**Keywords:** restrained eating, response inhibition, stop-signal task, implicit associations, cognitive training, inhibitory control

## Abstract

Restrained eaters display difficulties engaging in self-control in the presence of food. Undergoing cognitive training to form associations between palatable food and response inhibition was found to improve self-control and influence eating behaviors. The present study assessed the impact of two such response inhibition trainings on food consumption, food-related anxiety, and implicit attitudes toward food among female restrained eaters (Dutch Eating Behavior Questionnaire-restrained eating subscale ≥ 2.5). In Experiment 1, 64 restrained eaters completed either one of two training procedures in which they were asked to classify food vs. non-food images: a food-response training, in which stop cues were always associated with non-food images, or a balanced food-response/inhibition training, in which participants inhibited motor actions to food and non-food stimuli equally. The results revealed reduced snack consumption following the food-response/inhibition training compared to the food-response training. The food-response training was associated with increased levels of food-related anxiety. In Experiment 2, the same training procedures were administered to 47 restrained eaters, and implicit attitudes toward palatable foods were assessed. The results revealed an increase in positive implicit attitudes toward palatable foods in the food-response/inhibition group but not in the food-response training group. The results suggest that balancing response inhibition and execution across food and non-food stimuli may reduce overeating while retaining positive attitudes toward food among female restrained eaters.

## 1. Introduction

Restrained eaters, or “chronic dieters”, are individuals who restrict food intake in an attempt to promote weight loss or avoid gaining weight [1]. Restrained eaters are generally highly motivated to restrict their food intake in order to control body weight. Paradoxically, several studies have shown that restrained eaters consume larger amounts of food compared to unrestrained eaters [2,3]. Additionally, severe restrained eating is a significant risk factor for eating disorders and is associated with increased levels of depression and anxiety [4]. As such, it is important to better understand the underlying factors that affect restrained eating and how to promote more balanced eating patterns. 

Exposure to palatable high-calorie foods is one of the common risk factors for overeating among restrained eaters (for review see [5]). Difficulty engaging in self-control while being exposed to palatable foods may be a consequence of a failure to activate neurocognitive abilities, such as response inhibition. Response inhibition is an executive function which allows one to pursue goal-directed behavior by overriding actions or thoughts based on a strong internal predisposition or external lure [6]. Indeed, multiple studies have demonstrated an association between response inhibition and eating behaviors (for review see [7]). Several researchers have suggested that inefficient activation of response inhibition, especially following exposure to food stimuli, may reflect difficulties in self-control and maintain binge eating episodes in individuals with bulimia nervosa, binge eating disorder, and obesity [8,9,10,11,12]. In contrast, superior response inhibition triggered following the presentation of high-calorie foods has been demonstrated in patients with anorexia nervosa [13], a disorder characterized by dangerous weight loss due severe dietary restraint, fear of gaining weight, and body image disturbance [14]. Specifically, over-activation of response inhibition was hypothesized to allow patients with anorexia nervosa to endure prolonged periods of self-starvation [13,15]. Taken together, it seems that imbalanced activation of response inhibition (i.e., inefficient/under-activation or superior/over-activation) may underlie various disordered eating styles (i.e., overeating and restricted eating, respectively). 

Restrained eating is not a psychiatric disorder as eating disorders are. However, dietary restraint is a core feature in eating disorders such as bulimia and anorexia nervosa [14]. Thus, studying response inhibition among healthy individuals with restrained eating may shed light on the phenomenon, independently of comorbid psychopathologies and physical complications that are commonly associated with eating disorders. Imbalanced activation of response inhibition was also reported in nonclinical samples of restrained eaters [16,17,18]. For example, in a previous study, we showed that restrained eaters were better at inhibiting a response following exposure to palatable food images compared to non-food images [18]. However, when being exposed to neutral non-food stimuli, restrained eaters’ response inhibition abilities were poorer compared to that of unrestrained eaters [18]. This pattern suggests that a strong activation of response inhibition following exposure to food stimuli may support restrained eaters’ goal to reduce food consumption. However, in the long run, due to a general deficit in inhibitory resources, such restriction may lead to a paradoxical breakdown of control over eating behaviors [3]. Again, this pattern strengthens the notion that imbalanced activation of response inhibition abilities may be involved in disordered eating among restrained eaters. Taken together with the evidence reviewed above, it is not surprising that studies found that training response inhibition can directly influence eating behaviors (for reviews see [19,20]). 

Computerized training procedures that train response inhibition to food usually involve associating images of palatable foods with stopping by presenting images of food along with task cues that instruct stopping an action. The association formed between palatable foods and stopping was shown to reduce food consumption among healthy individuals and those with obesity [19,20]. Similar training interventions were used with other clinical populations in which a stimulus that commonly triggers unwanted maladaptive behavior (e.g., compulsions in obsessive–compulsive disorder) was associated with response inhibition in order to extinguish compulsive behaviors [21]. Interestingly, associating stop cues with palatable food images influences not only eating but also attitudes toward palatable foods among healthy individuals and those with obesity. For example, in a series of studies, Chen and colleagues have shown that palatable food stimuli are rated as less attractive after a training task that associated palatable food images with stop cues [22,23,24]. 

To date, most studies have attempted to reduce food consumption and create negative attitudes toward food among different populations by associating response inhibition with food stimuli. However, it seems that a more therapeutic goal for restrained eaters would be to achieve greater balance between response inhibition and response execution in the presence of food, rather than training them to constantly stop their responses in the presence of palatable foods. In other words, self-control in the presence of food should reflect flexibility between food consumption and restriction—an ability that seems to be lacking in restrained eaters. Improving self-control in the presence of food in such a way may also reduce food-related anxiety and increase positive attitudes toward food among individuals who chronically restrict food intake. 

The goal of the present study was to assess the impact of two response inhibition training procedures on food consumption, food-related anxiety, and implicit attitudes toward palatable foods among female restrained eaters. In one training group, restrained eaters completed a behavioral task in which palatable food images were always associated with response execution and non-food images with response inhibition. That is, restrained eaters never had to inhibit their response upon seeing food and always had to inhibit their response when seeing non-food stimuli (i.e., food-response group). In a second training group, the task was modified so that restrained eaters had to inhibit their response to food and non-food images in an equal proportion (i.e., food-response/inhibition group). The primary outcome measures were snack consumption in a bogus taste test, changes in food-related anxiety (Experiment 1) and implicit attitudes toward palatable foods in the food–valence compatibility task (Experiment 2) following the training. We expected that snack consumption will be smaller in the balanced food-response/inhibition training group compared to that in the food-response training group. Additionally, we expect that the food-response/inhibition training will result in reduced food-related anxiety and an increase in positive implicit attitudes toward high-calorie foods. 

## 2. Experiment 1

### 2.1. Method

#### 2.1.1. Participants

Sixty-eight restrained eaters participated in this experiment in return for a small monetary reward. Because restrained eating as a means to control weight is far more common in females than in males, only female restrained eaters were recruited for the current study. Demographics and characteristics of the sample are shown in Table 1. All participants had normal or corrected-to-normal vision, had no history of attention deficits or dyslexia, and were unaware of the purposes of the experiment. All participants were recruited from an undergraduate university sample. Potential participants were initially screened using an online survey, which included the Restrained Eating subscale of the Dutch Eating Behavior Questionnaire (DEBQ-R) [25]. Participants who scored >2.5 were invited to the lab by email to participate in the study. Body mass index (BMI) lower than 18.5 or greater than 35 was used as an exclusion criterion. Four participants did not complete the bogus taste test, and therefore, their results were not further analyzed (three refused to taste at least one of each snack and one was fasting due to a religious holiday). The final sample included 64 female participants. The participants were randomly assigned to either the food-response training group or the balanced food-response/inhibition training group (Table 1). 

#### 2.1.2. Procedure

The study was approved by the ethical committee of the Hebrew University of Jerusalem and followed the American Psychological Association (APA) ethical standards. To reduce differences in a priori states of hunger, participants were asked to refrain from eating or drinking anything but water 3 h prior to the experiment. After signing an informed consent form, participants completed the following steps (a) participants completed self-report measures, including the DEBQ-R and questions regarding their hunger (“How hungry are you at the moment from 1—Not hungry at all to 10—Extremely hungry”), weight, and height, and were asked to assess their current level of food-related anxiety using a visual analog scale (VAS; “How anxious are you right now about issues related to food and eating”). (b) Participants were randomly assigned into one of the two groups: the food-response or the food-response/inhibition training group. Based on the training group, each group completed a different version of the food stop-signal task (F-SST) [18] (further details below). (c) Following the task, participants completed a bogus taste task to measure food consumption. (d) Finally, participants were asked to answer self-report questions, identical to those asked at baseline, regarding their hunger and current level of food-related anxiety (using a VAS). 

In order to ensure that participants were unaware of the purpose of the experiment, the taste test and the F-SST were presented as two separate studies. Participants were told that they were recruited for a laboratory taste test but were told that they will have to wait 15 minutes after completing the questionnaires and before the taste test. Then, they were offered to “use this time” to participate in “a different study in our lab” (the F-SST) for additional payment. All participants agreed. Participants received a total of ~10 USD (5 USD for the “original” taste test and another 5 USD for the “additional” computerized task). 

#### 2.1.3. Measures

The Restrained Eating Subscale of the Dutch Eating Behavior Questionnaire (DEBQ-R) [25]. The restrained eating subscale includes 10 questions regarding one’s tendency to restrict food consumption. Ratings are made on a five-point Likert scale. Participants completed the Hebrew version of the questionnaire. The questionnaire was translated to Hebrew in the following way: Two independent translators translated the questionnaire to Hebrew. Inconsistencies were then discussed. Next, a third translator reverse-translated the questionnaire back to the English to ensure clear understanding of all items. Cronbach’s alpha value in the original study was 0.95 [25] and in the current study it was 0.89.

**Figure 1:**
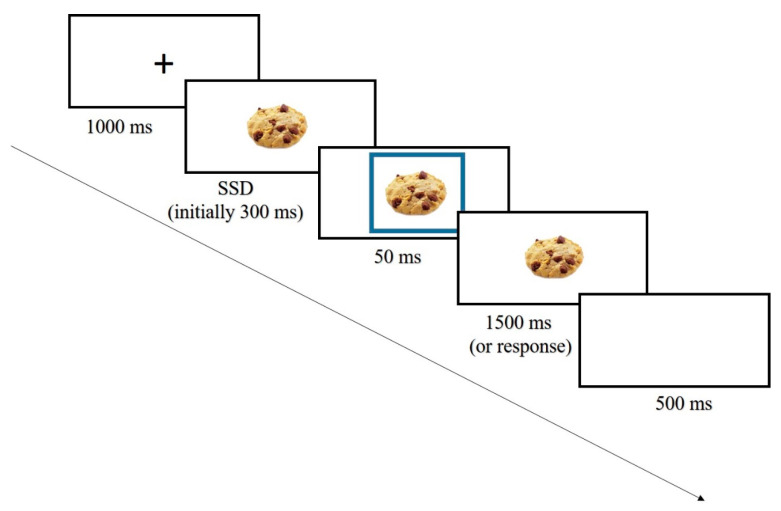
An example of a stop-food trial in the F-SST (food stop-signal task).

The Food Stop-Signal Task (F-SST; Figure 1). The two versions of the F-SST were administered to associate food/no-food stimuli with either response or stopping behaviors. Each trial in the task started with a black fixation point presented at the center of a white screen for 1000 ms. Next, participants were shown an image of either a food or non-food item in the center of the screen (i.e., a go signal). Participants were instructed to press the “z” key on the keyboard for food stimuli or the “?” key for non-food stimuli as fast as possible. Forty images of food (18 sweet and 22 savory) and 40 non-food images (household items) were selected from the “food pics” database [26]. On a random selection of 25% of the trials, a stop signal (i.e., a blue frame that appeared for 50 ms) was presented after a “stop-signal delay” (SSD) of 300 ms. Participants were instructed to withhold their response upon seeing the stop signal. Each trial ended with a 500 ms inter-trial interval. The task started with 32 practice trials that included feedback on accuracy and response times (RTs). The experimental task included 240 trials. 

In order to create two training groups, the proportion of trials in which a stop signal followed food or non-food stimuli was manipulated. In the food-response training group, all 60 trials that included a stop signal were trials in which the stop signal appeared after non-food images and never after food items. That is, this task always involved executing a behavioral motor response when being exposed to food images. In the food-response/inhibition training group, the stop signals were distributed equally across trials, which included food and non-food images as go-signals (i.e., 30 stop signals on food trials and 30 on non-food trials), creating a balance between response inhibition following exposure to food and non-food items. 

The bogus taste test [27] was used to measure food consumption. Three bowls of palatable snacks containing chocolate-covered peanuts (M&Ms; 3.4 kcal each), hazelnut biscuits (Loacker; 17.4 kcal each), and pretzel sticks (5 kcal each) were presented to each participant (all snacks were about the same size). Note that none of these snacks were used in the F-SST task. Participants were asked to “taste the snack from each bowl” (in a consistent order) and were asked to rate the taste of each snack on a scale of 1 to 10. No instruction was given regarding the amount food that the participant needed to taste (tasting at least 1 snack from each of the three bowls was mandatory in order to participate in the study). After each participant, the bowls were collected, and the total amount of snacks eaten was recorded.

### 2.2. Results

As presented in Table 1, there were no differences between the two groups of restrained eaters in age, BMI, and DEBQ-R. Before assessing differences in food consumption between the groups, we conducted a t-test to assess differences in hunger level between the groups before the training and found no differences in hunger level between the food-response training group (mean = 4.47, SD = 2.23) and the food-response/inhibition training group (mean = 3.97, SD = 1.77) (*t*(62) = 0.99, *p* = 0.324).

To ensure proper task engagement, we calculated two measures for the F-SST task: the nsRT (RT in no-stop-signal trials) and the nsACC (accuracy in no-stop-signal trials). There were no significant differences between the groups in both nsRT (food-response training: mean = 540, food-response/inhibition training: mean = 527; *t*(62) = 0.71, *p* = 0.480) and nsACC (food-response training: mean = 0.96, food-response/inhibition training: mean = 0.95; *t*(62) = 0.55, *p* = 0.585), indicating similar task engagement in both groups, on measures that are not affected by the specific version of the task.

**Figure 2:**
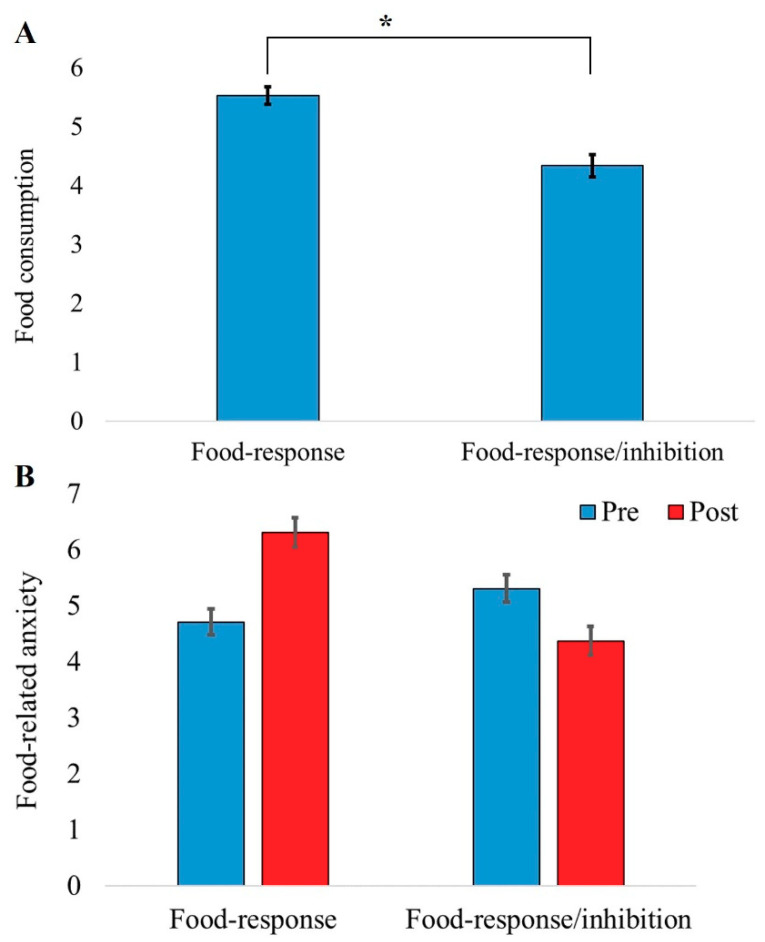
(**A**) Differences in snack consumption between the food-response and the food-response/inhibition training groups. The *y-*axis represents the total number of snacks eaten from the three bowls. (**B**) Changes in food-related anxiety as a function of training group and time. The *y*-axis shows the food-related anxiety score on the visual analog scale (VAS). The *x*-axis represents group. Error bars represent 1 standard error from the mean. * indicates *p* < 0.05.

Next, food consumption was calculated for each participant as the sum of snacks tasted from all three bowls. To test our primary hypothesis, independent t-tests were carried out to assess differences between the training groups in food consumption. As was hypothesized, the results showed reduced food consumption in the food-response/inhibition training group compared to the food-response training group. This was evident both in number of snacks eaten (5.53 (SE = 0.39) in the food-response group vs. 4.34 (SE = 0.29) in the food-response/inhibition group*; t*(62) = 2.45, *p* = 0.017, Cohen’s d = 0.61; Figure 2A) and in the total amount of kcal consumed (48.88 (SE = 4.23) in the food-response group vs. 37.34 (SE = 3.27) in the food-response/inhibition group; *t*(62) = 2.16, *p* = 0.034, Cohen’s d = 0.54).

In order to assess changes in food-related anxiety following the trainings, we conducted a two-way mixed model (ANOVA) on food-related anxiety score on the VAS with time (pre vs. post) as a within-subject factor and group (food-response training vs. food-response/inhibition training) as a between-subject factor. There were no main effects for time (*F*(1, 62) = 0.37, *p* = 0.547) or group (*F*(1, 62) = 2.08, *p* = 0.154). Importantly, the group × time interaction was significant (*F*(1, 62) = 5.46, *p* = 0.023, η_p_^2^ = 0.08). Planned comparisons revealed a trend toward increased food-related anxiety from pre- to post-training in the food-response training group (*t*(31) = 1.91, *p* = 0.06, Cohen’s d = 0.56; Figure 2B), whilst in the food-response/inhibition group, there was a reduction in food-related anxiety from pre- to post-training, but this did not reach significance level (*t*(31) = 1.36, *p* = 0.18; Figure 2B).

### 2.3. Discussion Experiment 1

In line with the a priori hypothesis, the results showed reduced food consumption in the food-response/inhibition training group compared to that in the food-response training group. This supports the notion that a response inhibition training aimed to balance response execution and inhibition to food stimuli can influence actual food consumption among female restrained eaters. In addition, differences between the training groups were observed in food-related anxiety. Specifically, a trend showing an increase in food-related anxiety following the food-response training and reduced food-related anxiety following the food-response/inhibition training resulted in a significant interaction between time and training group. That said, the simple effects testing pre- and post-training changes in each group separately did not reach a significance level. Nevertheless, the interaction between time and group showed that food-specific response inhibition training can influence not only eating behaviors but also the emotional response to food. This is important because previous studies have shown that restrained eaters tend to overeat in response to negative emotions such as stress [28]. High-calorie food stimuli, such as those used in the training task, are considered threat-provoking stimuli for restrained eaters because high-calorie foods are associated with weight gain. Thus, the fact that the food-response/inhibition training exposed participants to palatable high-calorie foods yet reduced food-related anxiety is clinically meaningful. While Experiment 1 implies that a balanced food-response/inhibition training can help prevent overeating and change food-related anxiety, it does not inform us regarding the participants’ attitudes toward food. Previous studies have shown that following response inhibition trainings, palatable foods are often rated as less attractive [24]. Therapeutically, encouraging negative attitudes toward food among restrained eaters may not be ideal since these individuals already hold more negative attitudes toward food compared to unrestrained eaters [29]. Therefore, Experiment 2 was conducted to assess whether and how a balanced food-response/inhibition training influences restrained eaters’ implicit attitudes toward food stimuli. 

## 3. Experiment 2

### 3.1. Method

#### 3.1.1. Participants

Fifty-three female restrained eaters, who did not participate in Experiment 1, participated in this experiment in return for small monetary compensation (~5 USD). Inclusion and exclusion criteria were identical to Experiment 1. The study was administered online. Six participants were excluded from further analyses due to low task engagement: five due to less than 70% accuracy in the food–valence compatibility task (FVCT; either before or after training) and one due to an extremely high number (> 2.5 SD) of no response trials. The final sample therefore included 47 female participants. The participants were randomly assigned to either the food-response training (*n* = 23) or the food-response/inhibition training (*n* = 24) group. Demographic and clinical characteristics of the two groups are presented in Table 1.

#### 3.1.2. Procedure

**Figure 3:**
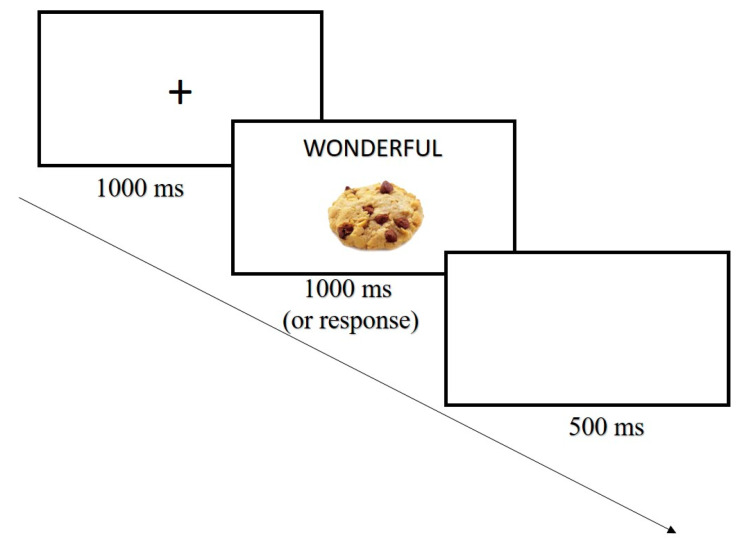
An example of a positive food trial of the food–valence compatibility task (FVCT) used in Experiment 2. Participants are asked to classify pleasant words to a “positive” category and unpleasant words to a “negative” category.

The study was approved by the Institutional Review Board of the Hebrew University of Jerusalem and followed APA ethical standards. Procedures in Experiment 2 were almost identical to those of Experiment 1. In Experiment 2, the SSD in the food-stop-signal task was initially set to 300 ms and a tracking procedure was then applied (see [30]). The only differences were that participants completed the FVCT (Figure 3) before and after completing the F-SST in order to assess changes in their implicit attitudes toward palatable foods, and that the taste test was not administered.

#### 3.1.3. Measures

The food–valence compatibility task (FVCT; Figure 3) was used to assess implicit attitudes toward food by examining response interference caused by associating palatable food images with positive and negative words. Each trial of the task began with a 1000 ms fixation followed by the target word that was presented for 1000 ms or until response. In each trial, one of four clearly positive words (i.e., excellent, wonderful, great, or pleasurable) or one of four negative words (i.e., gross, disgusting, terrible, or horrifying) was randomly selected and presented at the upper center panel of the screen. Under the target words, prime-pictures were presented. The prime-pictures were randomly selected out of 10 palatable food and 10 non-food pictures. Participants were asked to categorize the words to positive vs. negative-valence words by pressing the “Z” key (with their left hand) or the “?” key (with their right hand), respectively. Instructions emphasized the need to respond to the target word as quickly and as accurately as possible. Prior to the experimental block, a training block was administered. This training block was used in order to train participants to associate a left response with positive words and a right response with negative words. The training block was identical to the experimental block but consisted of the word “good,” that appeared in the upper left side of the screen, and the word “bad,” that appeared at the upper right side of the screen. The prime-pictures were not presented in the training block, and participants received feedback for response time (RT) and accuracy. The task therefore started with a block of 12 random training trials followed by an experimental block of 80 trials. 

The main dependent measure of the FVCT was the food–valence association effect, which is calculated as RT for non-food trials minus RT for food trials. The food–valence association effect is calculated separately for each valence condition (positive vs. negative). In the negative-valence condition, a larger effect (non-food RT > food RT) indicates that food and negative words are associated and thus suggests more negative attitudes toward palatable food, whilst in the positive-valence condition, a larger effect (non-food RT > food RT) indicates that food and positive words are associated and thus suggests more positive attitudes toward palatable foods. 

### 3.2. Results

As presented in Table 1, there were no differences between the two groups of restrained eaters in age, BMI, and DEBQ-R. Mean reaction time (RT) in the FVCT was calculated for each participant. The food–valence association effect was calculated for each participant in each valence (negative vs. neutral) and time (pre- vs. post-training) condition.

To ensure proper task engagement, we calculated two measures for the F-SST task: the nsRT (RT in no-stop-signal trials) and the nsACC (accuracy in no-stop-signal trials). There were no significant differences between the groups in both nsRT (food-response training: mean = 592, food-response/inhibition training: mean = 583; *t*(45) = 0.33, *p* = 0.740) and nsACC (food-response training: mean = 0.93, food-response/inhibition training: mean = 0.94; *t*(45) = 1.40, *p* = 0.166), indicating similar task engagement in both groups.

In order to validate our measurement of implicit association toward food (food–valence association effect), we tested whether this effect at baseline (prior to practice) correlated with the DEBQ-R scores. To that end, we subtracted the food–valence association effect for the negative valence from the food–valence association effect for the positive valence (i.e., a more positive attitude toward food or a less negative attitude toward food will result in a larger estimate). Results yielded a significant correlation between this estimate and DEBQ-R scores at baseline (*r*(46) = -0.31, *p* = 0.035), indicating that highly restrained eaters will exhibited more negative and less positive attitudes toward food.

**Figure 4:**
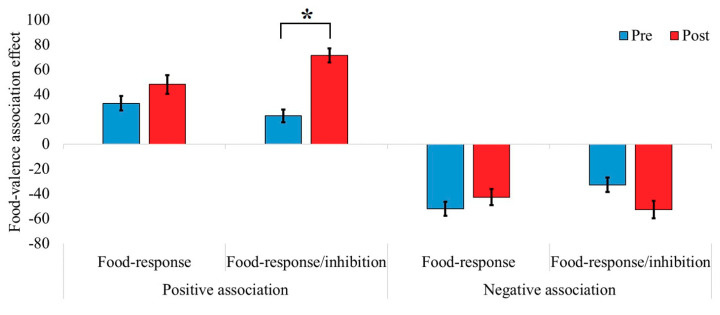
Differences in the food–valence association effect as a function of group, time, and valence. The food–valence association effect was calculated as mean response time (RT) in the non-food condition minus that in the food conditions. On the left panel (positive-valence condition) higher scores indicate greater positive associations whilst on the right panel (negative-valence condition) higher scores indicate greater negative associations. Error bars represent 1 standard error from the mean. * indicates *p <* 0.05.

A three-way mixed model ANOVA was carried out on the food–valence association effect, with group (food-response vs. food-response/inhibition training) as a between-subject factor, and valence (negative vs. positive) and time (pre-training vs. post-training) as within-subject factors (Figure 4 and Table 2). The results revealed a significant main effect for valence, (*F*(1, 45) = 58.57, *p* < 0.001, η_p_^2^ = 0.57), indicating a larger food–valence association effect for the positive compared to negative valence condition. There was also a significant main effect for time (*F*(1, 45) = 5.40, *p* = 0.025, η_p_^2^ = 0.11), indicating a larger food–valence association effect pre-training (mean effect = −7.3, SD = 34.3) compared to post-training (mean effect = 6.0, SD = 22.9). The main effect for group was not significant (*F*(1, 45) = 2.58, *p* = 0.116). Importantly, the three-way interaction between group, valence, and time was significant, (*F*(1, 45) = 5.12, *p* = 0.029, η_p_^2^ = 0.10). To further investigate this interaction, planned comparisons were carried out to examine the effects of time and group for each valence condition separately. In the positive associations condition, the results showed a significant increase in the food–valence association effect (i.e., an increase in implicit positive attitudes toward food) pre- to post-training in the food-response/inhibition training group (*F*(1,44) = 13.052, *p* < 0.001, η_p_^2^ = 0.22), but not in the food-response training group (*F*(1,44) = 1.094, *p* = 0.301, η_p_^2^ = 0.02). In the negative association condition, there were no pre to post differences in the food–valence association effect in the food-response/inhibition group (*F*(1,44) = 2.257, *p* = 0.14, η_p_^2^ = 0.04), nor in the food-response training group (*F*(1,44) = 1.21, *p* = 0.276, η_p_^2^ = 0.02) (Figure 4 and Table 2).

### 3.3. Discussion Experiment 2

The results of Experiment 2 revealed an increase in positive implicit attitudes toward palatable foods following the food-response/inhibition training, but not following the food-response training. There were no differences between the training groups in negative implicit attitudes toward food. Previous studies have shown that inhibiting motor responses while being exposed to food stimuli leads to a food devaluation effect. Specifically, inhibited food stimuli were rated as less attractive following such training procedures [23,24]. Our results showed that balanced response inhibition training can increase, rather than reduce, positive implicit associations regarding food stimuli among female restrained eaters. This is especially important considering that restrained eaters already hold negative attitudes toward food [29]. Therefore, finding ways to increase positive attitudes toward food among these individuals, while retaining self-control, is clinically important. 

## 4. Discussion

The current study compared two response inhibition trainings on food consumption, food-related anxiety (Experiment 1), and implicit attitudes toward food (Experiment 2) among female restrained eaters. Results yielded that a food-response/inhibition training that balanced the requirement to inhibit and respond to food and non-food stimuli reduced food consumption and increased positive implicit attitudes toward food. On the other hand, a food-response training procedure that encouraged a response to food stimuli and inhibition to non-food stimuli increased food-related anxiety and had no influence on implicit attitudes toward food. 

The current study contributes to the existing literature in several important ways. First, response inhibition trainings are currently being investigated as means for influencing food consumption. However, most studies form consistent associations between food and stopping a response [19,20]. Besides reduction of food consumption, such training procedures also elicit negative attitudes toward food among participants [22,23,24]. In contrast to those studies, the present study showed that a response inhibition training procedure that balances the requirement to stop a response to food and non-food stimuli reduces food consumption but also improves implicit attitudes toward food. This finding is clinically important because therapeutically, we would like to help restrained eaters achieve more flexible eating behaviors while retaining positive attitudes toward food (i.e., not experiencing food as a threat). Second, previous studies have shown that restrained eaters have a general deficit in response inhibition to non-food stimuli [16,17,18] and exert more inhibitory resources in order to inhibit their response to food stimuli compared to non-restrained eaters [18,31]. It has been postulated that in the long run, the exhaustion of inhibitory resources may subsequently lead to disinhibited eating behaviors such as overeating [3]. These findings suggest that overeating among restrained eaters may be the result of a lack of balance between over-activation and under-activation of inhibitory reactions to food and non-food stimuli. The present study supports this theory by showing that self-control over eating may be achieved by training restrained eaters to balance response inhibition and execution between food and non-food stimuli. Our results indicate that such balancing may improve restrained eaters’ self-control in the presence of food. 

In a broader perceptive, the current study adds to the mounting evidence suggesting that response inhibition is a modular process that can be trained and result in real-life behavioral changes. Specifically, individuals differ in their ability to use response inhibition as was previously indicated in behavioral and imaging studies [32,33,34]. Individual differences in response inhibition mean that some individuals experience marked difficulties recruiting inhibitory resources in everyday situations. As noted earlier, general response inhibition failures have been documented among restrained eaters, which may result in difficulty engaging in self-control in the presence of palatable foods [16,18]. Nevertheless, as the current study and others demonstrate, the modular nature of response inhibition allows training an individual to stop an automatic response in the presence of specific environmental cues. Indeed, previous studies have shown that conditioning response inhibition to activate during exposure to specific-environmental cues such as beer, cigarettes, and chocolate in lab-based experiments can subsequently alter how the trained individual behave when presented with these type of cues in real-life situations [35,36,37].

Increasing the knowledge on how response inhibition interacts with environmental cues and how it can be trained may have implications on various psychological disorders that are characterized by stimulus-driven behaviors or impulse-control problems. In fact, recent studies have provided preliminary reports that such conditioning can be beneficial in augmenting treatments for clinical populations (e.g., [38]). For example, in a recent study, a modified version of the stop-signal task was used to condition automatic inhibition in treatment of refractory patients with obsessive–compulsive disorder [21]. This study demonstrated that associating disorder-specific stimuli with stopping can not only change behaviors but also reduce unwanted intrusive negative cognitions. Similarly, the training procedures used in the current study, not only influenced food intake but also had an effect on food-related anxiety and attitudes toward food. Other studies have also shown that response inhibition can result in attitudinal changes regarding food cues that were associated with stopping a response [22,23,24]. Taken together, converging evidence suggest that response inhibition trainings can act to regulate emotions, thoughts, and behaviors. 

With respect to clinical implications, although restrained eating is not considered a psychiatric disorder, it is associated with elevated levels of depression and anxiety [4]. Moreover, studies have reported that dietary restraint is a proxy for developing eating disorders such as binge eating disorder and bulimia nervosa (for review see [39]). Restrained eating is also associated with weight gain and obesity [40]. Therefore, there is great importance in identifying ways to improve restrained eaters’ control over eating while retaining positive attitudes toward food. Response inhibition training such as that tested in the current study may, in the future, show promise as a means to regulate disordered eating patterns among individuals who are at high risk for developing eating disorders. Nevertheless, the field of cognitive training using appetitive food cues is relatively new. There are still many questions that require answers before such training can be offered as clinical interventions. For example, it is still not clear whether response inhibition trainings using appetitive cues have a lasting effect. Additionally, the exact amount of training sufficient for eliciting long-term effects is yet to be determined. Lastly, most response-inhibition trainings using appetitive food cues focus on changing eating behaviors. As such, there is still much to learn regarding the impact of such trainings on emotional and attitudinal factors that can be clinically meaningful and influence one’s emotional experience during exposure to various foods. In the future, it would also be interesting to assess such training procedures as potential add-on treatments for eating disorders in which over- and under-activation of response inhibition in the presence of food represent core clinical symptoms of the disorders such as self-starvation in anorexia nervosa and binge eating in bulimia nervosa and binge eating disorder [8,13].

Several limitations of the current study should be addressed. Experiment 1 did not include a baseline measurement of food consumption so that the purpose of the training would not be revealed. However, a lack of baseline food consumption measurement makes it difficult to determine whether the difference found in snack consumption between the training groups is because of a reduction of food intake in the food-response/inhibition group or an increase in food intake in the food-response training group. Future studies should include a baseline measurement of food intake or include a third control group that does not perform any training. Nevertheless, the results showed that there were no baseline differences between the groups in hunger level. A second limitation is that Experiment 2 was run online using a modest sample. Our initial plan was to replicate the results on food consumption and add the implicit attitude measures, but due to COVID-19, we could not conduct a lab-based experiment with a taste test. Thus, future studies will need to replicate the results with a larger sample in order to affirm the beneficial role of the food-response/inhibition task on food consumption and implicit attitudes toward food. Finally, the DEBQ-R does not have a standardized threshold for defining high-restrained eating. Therefore, it could be that other thresholds than that used in the current study would yield different results. However, it is important to note that the same threshold was used in both training groups as the study only tested high-restrained eaters.

To conclude, the current study revealed that response inhibition training that balances the requirement to stop a response to food and non-food stimuli can reduce food consumption and improve positive attitudes toward food among restrained eaters. This study adds to the existing knowledge regarding how eating behaviors can be modulated using cognitive training procedures that target neurocognitive mechanisms suggested to underlie disordered eating. Future studies should investigate the utility of such training procedures as intervention programs with a goal to achieving long-term effects on eating-related thoughts, emotions, and behaviors. 

## Figures and Tables

**Table 1:** Characteristics of training and control groups.

**Experiment 1**				
**Factor**	**Food response** **(*n* = 32)**	**Food response/inhibition (*n* = 32)**	***t***	***p-*** **value**
Age	24.06 (2.4) [19–32]	24.13 (2.2) [19–31]	*t*(62) = 0.11	0.914
BMI	23.85 (3.3) [18.4–30.1]	24.16 (4.1) [15.2–35]	*t*(62) = 0.34	0.736
DEBQ-R	3.07 (2.88) [2.6–3.6]	3.08 (0.3) [2.6–3.6]	*t*(62) = 0.13	0.898
**Experiment 2**				
	**Food response** **(*n* = 23)**	**Food response/inhibition (*n* = 24)**		***p-*** **value**
Age	25.04 (4.1) [20–40]	26.54 (6.6) [20–49]	*t*(45) = 0.94	0.354
BMI	24.08 (3.7) [8.8–34.2]	25.33 (3.5) [20.3–35]	*t*(45) = 1.18	0.243
DEBQ-R	3.81 (0.5) [3.1–4.6]	3.78 (0.5) [3.1–5]	*t*(45) = -0.18	0.859

Mean, (SD), [Range] of sample characteristics. Independent t-test analyses for age, BMI (body mass index), and DEBQ-R (Restrained Eating subscale of the Dutch Eating Behavior Questionnaire reveal no differences between the groups.

**Table 2:** Results of Experiment 2—food–valence compatibility task (FVCT).

Factor	Positive Valence				Negative Valence			
	Pre-Training		Post-Training		Pre-Training		Post-Training	
	Non-food	Food	Non-food	Food	Non-food	Food	Non-food	Food
Food response/inhibition	646 (97) [0.91]	623 (93) [0.95]	645 (92) [0.84]	573 (79) [0.96]	614 (96) [0.95]	647 (111) [0.86]	560 (80) [0.97]	612 (98) [0.87]
Food response	621 (89) [0.87]	592 (76) [0.93]	613 (85) [0.87]	569 (78) [0.95]	569 (71) [0.97]	625 (84) [0.87]	558 (67) [0.97]	598 (86) [0.89]

Note: Mean reaction time, (SD), and [accuracy] for the different conditions of Experiment 2.
